# An Updated List of Generic Names in the Thoracosphaeraceae

**DOI:** 10.3390/microorganisms1010122

**Published:** 2013-11-01

**Authors:** Marc Gottschling, Sylvia Soehner

**Affiliations:** Department of Biology, Systematic Botany and Mycology, GeoBio-Center, University Munich, Menzinger Str. 67, D-80638 Munich, Germany

**Keywords:** coccoid cell, molecular systematics, morphology, phylogeny, taxonomy, thecate cell

## Abstract

Calcareous dinophytes produce exoskeletal calcified structures during their life history (a unique character among the alveolates) and are subsumed under the Thoracosphaeraceae as part of the Peridiniales. We provide a brief synopsis about the taxonomic history of the group, from the first descriptions of fossils in the 19th century through to the results of molecular phylogenetics studies undertaken during the past two decades. Delimitation and circumscription of the Thoracosphaeraceae are challenging, as they comprise both phototrophic (presumably including endosymbiotic) as well as heterotrophic (and even parasitic) dinophytes from marine and freshwater environments, respectively. However, calcareous structures are not known from all members of the Thoracosphaeraceae, and the corresponding species and groups are considered to have lost the capacity to calcify. Five years ago, a taxonomic list of 99 generic names assigned to the Thoracosphaeraceae was published, and we update this compendium with 19 additional names based on recent studies.

## 1. Historical Survey

Many dinophytes develop two distinct stages during their life history, namely a motile thecate cell and a non-motile coccoid cell. During the coccoid stage, the production of exoskeletal calcified structures is a distinct character trait exclusively found in a subordinate collective of the Peridiniales Haeckel, notably in the calcareous dinophytes [1]. Their thecate cells exhibit a more or less conserved arrangement of cellulose plates (*i.e.*, the tabulation) and are presumed to be haploid, while the coccoid cells are usually interpreted as hypnozygotes (*i.e.*, diploid stage: [2]). The calcareous cells are morphologically highly diverse. Various degrees of expressed tabulation may be retained (formerly described as “paratabulation”), which is frequently restricted to the archeopyle (aperture for germination). Calcareous dinophytes are well documented in the fossil record, and their diversity assessment has a complex and uncompleted history.

From a paleontological perspective, the first descriptions of organisms with a calcified shell and today assigned to the dinophytes go back to F.J. Kaufmann, although he considered Cretaceous †*Lagena sphaerica* F.J.Kaufmann and †*L. ovalis* F.J.Kaufmann as members of the foraminifers [3]. At the beginning of the last century, T. Lorenz acknowledged the distinctiveness of those forms from †*Lagena* G.Walker & Boys, and subsequently introduced the generic name †*Pithonella* T.Lorenz, with the type species †*Pithonella ovalis* (F.J.Kaufmann) T.Lorenz and retained them in the foraminifers [4]. It took further decades until G. Deflandre recognized the true dinophyte nature of corresponding fossils, although he interpreted them as calcareous thecate cells [5]. Thereafter, the majority of the calcareous dinophyte diversity described was subsumed under the name Calciodinellaceae Deflandre (alternatively Calciodinelloideae Fensome, F.J.R.Taylor, G. Norris, Sarjeant, Wharton & G.L.Williams) and has since resulted in many studies [6,7,8,9,10,11,12,13,14,15,16,17,18].

Cultivation experiments with extant calcareous dinophytes showed that the immotile coccoid and not the motile cell is usually calcified [19]. Moreover, cultivation of calcareous dinophytes demonstrated that the thecate cells hatching from the coccoid cells can partly be assigned to species, which have long been known by neontologists [e.g., *Scrippsiella trochoidea* (F.Stein) A.R.Loebl.]. The pioneering work of D. Wall and B. Dale [19] was thus the start of numerous studies investigating in more detail the developmental link between thecate and coccoid cells in the life history of particular calcareous dinophyte species [20,21,22,23,24,25,26,27,28]. As a result, thecate cells of most calcareous dinophytes exhibit homogenously an ortho-hexa-tabulation pattern identifying them as members of the Peridiniales, whereas the morphology of coccoid cells is particularly diverse in calcareous dinophytes.

Similarly to the Calciodinelloideae, it took more than half a century until *Thoracosphaera heimii* (Lohmann) Kamptner (initially described under the coccolithophore *Syracophaera* Lohmann [29]) was recognized as a (calcareous) dinophyte [30,31,32]. *Thoracosphaera* Kamptner differs from the majority of calcareous dinophytes in several respects: the motile cells are athecate, and the calcareous coccoid cells are dividing vegetatively. These differences were considered so fundamental that the Thoracosphaerales Tangen were established at the same taxonomic level as the Peridiniales [32], implying that *Thoracosphaera* is only distantly related to the Calciodinelloideae. This classification was also followed in the epochal work of Fensome and colleagues [33] and by subsequent authors.

**Figure 1:**
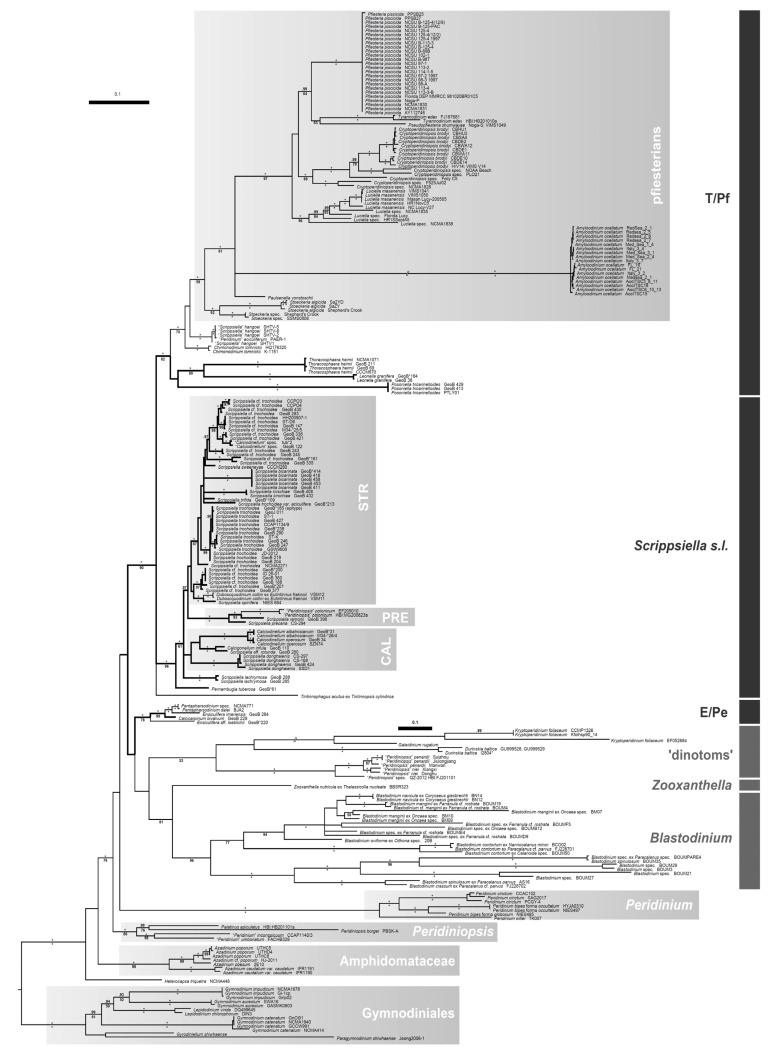
Phylogeny of and molecular delimitations in the Thoracosphaeraceae (Bayesian tree) segregating into the three indicated clades E/Pe, T/Pf, and *Scrippsiella s.l.*(abbreviations: CAL, †*Calciodinellum* and relatives; E/Pe, *Ensiculifera* + *Pentapharsodinium* and relatives; PRE, *Scrippsiella precaria* Montresor & Zingone and relatives; STR, *Scrippsiella trochoidea* species complex; T/Pf, *Thoracosphaera* + *Pfiesteria* and relatives). Calcareous taxa are highlighted by bold branches. Branch lengths are drawn to scale, with the scale bar indicating the number of substitutions per site. Numbers on branches are statistical support values (above: Bayesian posterior probabilities, values under 0.90 are not shown; below: ML bootstrap support values, values under 50 are not shown), and maximal support values are indicated by asterisks.

Since the onset of molecular studies, knowledge on the phylogenetic relationships and the constituent taxa of extant calcareous dinophytes has changed gradually but significantly overall. The early molecular studies identified two [23] and later three distinct evolutionary lineages [34] that include calcareous as part of peridinialean dinophytes (Figure 1), namely the E/Pe-clade (with species of *Ensiculifera* Balech and *Pentapharsodinium* Indel. & A.R.Loebl.), the T/Pf-clade (with species of *Thoracosphaera* and *Pfiesteria* Steid. & J.M.Burkh.), and *Scrippsiella* Balech *sensu lato* (*s.l.*, also including fossil-taxa such as †*Calciodinellum* Deflandre and †*Pernambugia* Janofske & Karwath). An important result of the molecular studies showed that *Thoracosphaera* is not distinct from other calcareous dinophytes but in fact embedded within them. It was therefore proposed to unify the formerly segregated taxonomic units Calciodinelloideae and Thoracosphaerales and to treat the entirety of calcareous dinophytes under the Thoracosphaeraceae J.Schiller [1].

Opposing the view of Tangen [32], various authors (with a predominantly paleontological background) considered calcareous dinophytes as a monophyletic group based on the apomorphic calcified coccoid cells [10,35]. However, the first molecular studies challenged this simplistic circumscription of calcareous dinophytes. The Thoracosphaeraceae included not only calcareous but also non-calcareous dinophytes or at least those of which calcareous structures are not known so far (Figure 1). The lack of calcified structures in those members of the Thoracosphaeraceae has been considered a secondary loss [1,34]. However, as more molecular studies were published non-calcareous dinophytes included in the Thoracosphaeraceae became greater in number and more heterogeneous, as outlined below.

The pfiesterians are a group of heterotrophic dinophytes and versatile predators. Some of their species have been associated with harmful algal blooms and fish kills, but many aspects of their life histories and character traits (e.g., potential toxin activity) are still under debate (see [36] and literature therein). Since its first description [37], the systematic position of *Pfiesteria* in the dinophyte tree was unclear and was placed somewhere in the Gonyaulacales F.J.R.Taylor or Peridiniales. It was thus a great surprise when molecular phylogenies identified calcareous *Leonella* Janofske & Karwath and *Thoracosphaera* as the closest known relatives of the pfiesterians and that the latter may derive from calcareous dinophytes [34]. This scenario has been repeatedly supported by subsequent studies (partly investigating alternative loci [28,38,39,40]), and the molecular trees indicate a single loss event of the capacity for calcareous structures in the T/Pf-clade (Figure 1). The acceptance of the Pfiesteriaceae as a distinct systematic unit [41,42,43,44] would, anyhow, leave the remainders of the Thoracosphaeraceae paraphyletic.

From an evolutionary perspective, the discovery of tintinnid parasites such as *Duboscquodinium* Grassé and *Tintinnophagus* Coats nested within the calcareous dinophytes of the *Scrippsiella s.l.* lineage based on molecular data [45] was presumably more unexpected than the *Pfiesteria* results. Moreover, K.D. Smith and colleagues reported that another ctenophoran parasite was closely related to †*Calcicarpinum bivalvum* G.Versteegh [= *Pentapharsodinium tyrrhenicum* (Balech) Montresor, Zingone & D.Marino] from the E/Pe-clade [46,47], highlighting the association between calcareous and parasitic dinophytes. However, it is presently unknown (and experimentally very difficult to investigate), whether calcareous dinophytes may exhibit also parasitic stages during their life history, in addition to the comparatively well investigated thecate and coccoid cells. The *Scrippsiella s.l.* lineage contains many species that are morphologically indistinguishable, but genetically differentiated (*i.e.*, cryptic species), which refers particularly to the *S. trochoidea* species complex [26,48,49]. Linking this cryptic diversity with the hypothetical specificity of parasitic dinophytes (as inferred from inoculation experiments [47]) has triggered the idea that the species of the *S. trochoidea* species complex are neither differentiated based on morphology or spatial distribution, but based on tight interactions with particular host species (*pers. comm.* K.J.S Meier, Kiel).

The close relationship between the T/Pf-clade and the *Scrippsiella* lineage is undisputed today, and the vast majority of extant calcareous dinophytes known is reliably placed in one of the two clades. However, the E/Pe-clade challenges the assumption of a monophyletic calcareous dinophyte group, as its close relationship to the *Scrippsiella*- and T/Pf-clades is not shown, or at least not supported, in all molecular studies. The only calcareous member of this clade with published sequence data is †*Calcicarpinum bivalvum*, while calcified structures are not known from any other sequenced species out of this clade assigned to *Ensiculifera* or *Pentapharsodinium*. However, the group is considered to include a number of calcareous, mostly fossil-taxa such as †*Follisdinellum* G.Versteegh, †*Melodomuncula* G.Versteegh, and *Pentadinellum* Keupp, all of which were observed in Recent sediments, but have not been brought in culture so far [1]. Exploring the extant diversity, and exact phylogenetic placement, of the E/Pe-clade thus remains one of the major tasks in future research on calcareous dinophytes.

The ambiguity of the phylogenetic position regarding the E/Pe-clade refers in particular to other peridinalean dinophytes, of which sequences have been published in the past few years. The molecular studies suggest the monophyly of a highly disparate group [28,50] comprising endosymbionts (*i.e.*, *Zooxanthella*), parasites (*i.e.*, *Blastodinium* [51,52]), and dinophytes harboring a diatom as endosymbiont (*i.e.*, the “dinotoms” [53,54,55]). In some molecular studies, this heterogeneous assemblage is closely related to the E/Pe-clade (*i.e.*, are members of the Thoracosphaeraceae [38,50], Figure 1), in others they constitute the sister group of the Thoracosphaeracae, which then consist of the three clades E/Pe, T/Pf, and *Scrippsiella s.l.* [40]. Currently, it has to be emphasized that molecular phylogenies of dinophytes still have room for improvement because of various problems, including limited taxon sampling (less than a quarter of dinophytes at the generic level are currently known with respect to genetic sequence data), insufficient genetic data, and strong rate heterogeneity (see discussion in [28]).

In summary, the often puzzling diversity of the Thoracosphaeraceae in terms of nutrition modes (phototrophic→heterotrophic), habitat preferences (marine→freshwater), and coccoid cell morphologies (calcareous→non-calcareous) reflects to some degree the variation found throughout all dinophytes. This biological heterogeneity makes a morphological diagnosis of the Thoracosphaeraceae almost impossible, and their taxonomic delimitation relies mostly on molecular data at present. Also, the taxonomy of the Thoracosphaeraceae is further challenging, as they have been described under the rules of the *International Code for Zoological Nomenclature* (ICZN [56]) as well as the *International Code of Nomenclature for algae, fungi and plants* (ICN [57]) and based on thecate as well as coccoid (and parasitic) stages (for details, see [1]). In their current circumscription, the Thoracosphaeraceae (Peridiniales, Dinophyceae) comprise about 70 extant (morpho-)species, plus about 260 fossil species. Within the impressive diversity of the Alveolata, the capacity to produce calcareous structures is restricted to (*i.e.*, has been considered apomorphic for) the Thoracosphaeraceae, arguing for the monophyly of the group [1,10,19]. The lack of calcified structures in those members of the Thoracosphaeraceae without known calcareous structures has then been considered a secondary loss [1,34].

## 2. Taxonomy

In the Agenda Calcareous Dinophytes from 2008, a list of 99 generic names in the Thoracosphaeraceae was published [1] based on the knowledge at that time. Since then, more taxa have been shown to be included in the Thoracosphaeraceae, and based on this work (Figure 1) and previous studies [45,58,59,60,61,62,63,64,65,66], the following 14 names are to be added to the list of generic names in the Thoracosphaeraceae (using the same reference format as in the Agenda Calcareous Dinophytes):

(1) ***Amyloodinium*** E.-M.Br. & Hovasse^Z^, *Proceedings of the Zoological Society of London* 116: 45. 1916. Type: ***Amyloodinium ocellatum*** (E.-M.Br.) E.-M.Br. & Hovasse^Z^, *l.c.*: 32–43, figs 1–9 ≡ ***Oodinium ocellatum*** E.-M.Br.^Z^, *Proceedings of the Zoological Society of London* 101: 345–346. 1931. Extant parasite in the gill mucosa of marine fish (without precise locality).

(2) †***Calciconus*** Streng, Banasová, D.Reháková & H.Willems^B^, *Review of Palaeobotany and Palynology* 153: 229. 2009 ≡ †*Trigonus* Banasová, Kopčáková & D.Rehaková^B^, not validly published (ICN Art. 36.1b). Type: †***Calciconus irregularis*** Streng, Banasová, D.Reháková & H.Willems^B^, *l.c.*: 230, pl. II 1–10 ≡ †*Trigonus conicus* Banasová, Kopčáková & D.Rehaková^B^, not validly published (ICN Art. 36.1b). Badenian (Slovak Republic: Bratislava).

(3) ***Chimonodinium*** Craveiro, Calado, Daugbjerg, Gert Hansen & Moestrup^B^, *Protist* 162: 604–605. 2011. Type: ***Chimonodinium lomnickii*** (Wołosz.) Craveiro, Calado, Daugbjerg, Gert Hansen & Moestrup^B^, *l.c.*: 605–606, figs 1–14 ≡ ***Peridinium lomnickii*** Wołosz.^B^, *nom. corr.* (ICN Arts 60.6, 60.12), *Bulletin International de l’Académie des Sciences de Cracovie, Classe des Sciences Mathématiques et Naturelles. Série B* 1915: 264, 267–268, pl. X 25–29. 1916 ≡ ***Glenodinium lomnickii*** (Wołosz.) Er.Lindem.^B^ in Schoen., *Einfachste Lebensformen des Tier- und Pflanzenreiches. Fünfte Auflage. Band 1 (Spaltpflanzen, Geißlinge, Algen, Pilze)*: 162, 168, 169. 1925. Extant (Ukraine: Lviv).

(4) †***Cylindratus*** Banasová, Kopčáková & D.Rehaková ex Streng, Banasová, D.Reháková & H.Willems^B^, *Review of Palaeobotany and Palynology* 153: 230. 2009. Type: †***Cylindratus borzae*** Banasová, Kopčáková & D.Rehaková ex Streng, Banasová, D.Reháková & H.Willems^B^, *l.c.*: 232, pl. III 1–9. Badenian (Slovak Republic: Bratislava).

(5) ***Duboscquodinium*** Grassé^Z^, *Traité de zoologie* 1: 358, 384. 1952. Type: ***Duboscquodinium collinii*** Grassé^Z^, *nom. corr.* (ICN Art. 60.12), *l.c.*: fig. 297A–B. Extant parasite (without precise locality).

Remark: If it can be reliably shown in future that *Dubosquodinium* and *Scrippsiella* are congeneric, then this would have dramatic consequences, as *Dubosquodinium* [67] is older than *Scrippsiella* [68] (even when A.R. Loeblich’s “validation” [69] is not considered) and would have taxonomic priority. However, further taxonomic activity should not be undertaken until the precise identity of the type species of *Scrippsiella*, *S. sweeneyae* Balech, is worked out.

(6) †***Juergenella*** Banasová, Kopčáková & D.Rehaková ex Streng, Banasová, D.Reháková & H.Willems^B^, *Review of Palaeobotany and Palynology* 153: 236. 2009. Type: †***Juergenella ansata*** (Hildebrand-Habel & H.Willems) Streng, Banasová, D.Reháková & H.Willems^B^, *l.c.*: 237 ≡ †***Calcigonellum ansatum*** Hildebrand-Habel & H.Willems^B^, *Journal of Micropalaeontology* 18: 93, pl. I 8–10. 1999. Upper Eocene (South Atlantic Ocean: Rio Grande Rise).

(7) ***Paulsenella*** Chatton^Z^, *Archives de Zoologie Experimentale et Generale* 59: 320. 1920. Type: ***Paulsenella chaetoceratis*** (Paulsen) Chatton^Z^, *l.c.*: fig. 139 ≡ ***Apodinium chaetoceratis*** Paulsen^B^, *Meddelelser om Grønland [11. Marine Plankton from the East-Greenland Sea 3]* 43: 316, fig. 17. 1910. Extant parasite (Atlantic Ocean: Greenland Sea).

(8) †***Posoniella*** Streng, Banasová, D.Reháková & H.Willems^B^, *Review of Palaeobotany and Palynology* 153: 233–234. 2009. Type: †***Posoniella tricarinelloides*** (G.Versteegh) Streng, Banasová, D.Reháková & H.Willems^ B^, *l.c.*: 234, fig. 5A,D,G ≡ †***Bicarinellum tricarinelloides*** G.Versteegh^B^, *Review of Palaeobotany and Palynology* 78: 357, 359–360, pl. I 4–5. 1993. Pleistocene (Greece: Crete).

(9) ***Stoeckeria*** H.J.Jeong, Jae S.Kim, J.Y.Park, Jong H.Kim, Sang Kim, I.Lee, Seung H.Lee, J.H.Ha & W.H.Yih^Z^, *Journal of Eukaryotic Microbiology* 52: 389. 2005. Type: ***Stoeckeria algicida*** H.J.Jeong, Jae S.Kim, J.Y.Park, Jong H.Kim, Sang Kim, I.Lee, Seung H.Lee, J.H.Ha & W.H.Yih^Z^, *l.c.*: 384–385, figs 1–23. Extant (Pacific Ocean: East China Sea, off Korea).

Remark: There is some discussion whether *Stoeckeria* was validly published (using botanical “Dinophyceae” in the title, but lacked a Latin description or diagnosis). We agree with our colleagues [62] that it was not the authors’ intention to publish the new name under the rules of the ICN and therefore accept it pragmatically as validly published under the rules of the ICZN.

(10) ***Theleodinium*** Craveiro, Pandeirada, Daugbjerg, Moestrup & Calado^B^, *Phycologia* 52. in press. Type: ***Theleodinium calcisporum*** Craveiro, Pandeirada, Daugbjerg, Moestrup & Calado^B^, *l.c.* Extant (Portugal: Gafanha da Boavista).

(11) ***Tintinnophagus*** Coats^Z^ in Coats, Su.Kim, Bachvaroff, Handy & Delwiche, *Journal of Eukaryotic Microbiology* 57: 481. 2010. Type: ***Tintinnophagus acutus*** Coats^Z^, *l.c.*: 471–473, figs 2–27. Extant parasite (USA–VA: Chesapeake Bay).

(12) †*Trigonus* Banasová, Kopčáková & D.Rehaková^B^, *Mineralia Slovaca* 39: 111. 2007, not validly published (ICN Art. 36.1b). Type: †*Trigonus conicus* Banasová, Kopčáková & D.Rehaková^B^, *l.c.*: 111–112, pl. I 9–12, not validly published (ICN Art. 36.1b). Badenian (Slovak Republic: Bratislava) ≡ †***Calciconus*** Streng, Banasová, D.Reháková & H.Willems.

(13) ***Tyrannodinium*** Calado, Craveiro, Daugbjerg & Moestrup^B^, *Journal of Phycology* 45: 1202–1203. 2009. Type: ***Tyrannodinium berolinense*** (Lemmerm.) Calado, Craveiro, Daugbjerg & Moestrup^B^, *l.c.*: figs 1–6 ≡ ***Peridinium berolinense*** Lemmerm.^B^, *Berichte der Deutschen Botanischen Gesellschaft* 18: 308–309. 1900 ≡ ***Glenodinium berolinense*** (Lemmerm.) Er.Lindem.^B^ in Schoen., *Einfachste Lebensformen des Tier- und Pflanzenreiches. Fünfte Auflage. Band 1 (Spaltpflanzen, Geißlinge, Algen, Pilze)*: 162, 164. 1925 ≡ ***Peridiniopsis berolinense*** (Lemmerm.) Bourr.^B^, *Protistologica* 4: 9. 1968. Extant (Germany: Berlin).

(14) P†***Zugelia*** Özdikmen^Z^, *Munis Entomology & Zoology* 4: 237. 2009. ≡ †*Normandia* Zügel^B^, not validly published (ICN Art. 53.1) (non: ***Normandia*** Hook.f.^B^, *Icones plantarum* 12: 20–21. 1872, nec: ***Normandia*** Pic^Z^, *Bulletin de la Société Entomologique de France* 1900: 267. 1900). Type: †***Zugelia circumperforata*** (Zügel) Özdikmen^Z^, *l.c.* ≡ †*Normandia circumperforata* Zügel^B^, *Courier Forschungsinstitut Senckenberg* 176: 32, 34, figs 12–13, pl. III 1–15. 1994, not validly published (ICN Art. 35.1). Turonian (France: Le Tilleul).

Remark: In the Agenda Calcareous Dinophytes [1], we overlooked that the name †*Normandia* Zügel had not been validly published neither under the ICN nor the ICZN because of the priority of earlier names. The affinity of the extinct pithonelloids to the calcareous dinophytes was debated in the past [1], but was recently corroborated based on exceptionally well preserved Cretaceous fossils [70].

The systematic position of a heterogeneous group comprising endosymbionts, dinophytes harboring endosymbionts, and parasites is not resolved at present with respect to the E/Pe-clade of the calcareous dinophytes. The following five names are therefore tentative candidates for being included in the Thoracosphaeraceae, but more research is necessary to determine their exact phylogenetic placement in the dinophyte tree:

(15) ***Blastodinium*** Chatton^Z^, *Comptes Rendus Hebdomadaires des Séances de l’Académie des Sciences* 143: 981. 1906. Type: ***Blastodinium pruvotii*** Chatton^Z^, *nom. corr.* (ICN Art. 60.12), *l.c.*: 981–983, figs 1–5. 1906. Extant (Mediterranean Sea, off France).

(16) ***Durinskia*** Carty & El.R.Cox^B^, *Phycologia* 25: 200. 1986. Type: ***Durinskia baltica*** (Levander) Carty & El.R.Cox^B^, *l.c.*: figs 7–14. 1986 ≡ ***Glenodinium balticum*** Levander^Z^, *Acta Societatis pro Fauna et Flora Fennica* 12.2: 52. 1894 ≡ ***Peridinium balticum*** (Levander) Lemmerm.^B^, *Kryptogamenflora der Mark Brandenburg. Dritter Band [Algen I (Schizophyceen, Flagellaten, Peridineen)]*: 657. 1910. Extant (Finland).

Remark: Although sporadically used, the species’ name has never been validly published under *Peridiniopsis* [71].

(17) ***Galeidinium*** Tam. & T.Horig.^B^, *Journal of Phycology* 41: 661. 2005. Type: ***Galeidinium rugatum*** Tam. & T.Horig.^B^, *l.c.*: 661–667, figs 1A–G, 2A–B, 3A–F, 4A–E. 2005. Extant (Western Pacific Ocean, off Palau).

(18) ***Kryptoperidinium*** Er.Lindem.^B^, *Botanisches Archiv* 5: 116. 1924. Type: ***Kryptoperidinium foliaceum*** (F.Stein) Er.Lindem.^B^, *l.c.*: 116–117, figs 12–20. 1924 ≡ ***Glenodinium foliaceum*** F.Stein^Z^, *Der Organismus der arthrodelen Flagellaten nach eigenen Forschungen in systematischer Reihenfolge bearbeitet* 2: pl. III 22–26 (1883). Extant (Baltic Sea, off Germany).

(19) ***Zooxanthella*** K.Brandt^B^, *Archiv für Anatomie und Physiologie/Physiologische Abteilung* 1881: 572. 1881. Type: ***Zooxanthella nutricula*** K.Brandt^B^, *l.c.* ≡ ***Endodinium nutricula*** (K.Brandt) A.Hollande & Carré, *nom. corr.* (ICN Arts 23.5, 32.2, ICZN Art. 32), *Protistologica* 10: 573–601. 1974. Extant (Mediterranean Sea, off Italy).

Remark: The species has also been placed under the generic names *Chrysidella* Pascher [72], *Amphidinium* Clap. & J. Lachm. [73], and *Scrippsiella* [74], but the corresponding combinations have never been validly published. R.J. Blank and R.K. Trench [73] discuss the nomenclature of endosymbiotic dinophytes in detail. Their proposal to reject the name *Zooxanthella* under the botanical code, however, has been rejected by the Committee for Algae [75]. Moreover, *Zooxanthella* is in need of proper typification [50].

General Remark: If the systematic placement of this group has been correctly determined among the calcareous dinophytes, then the names Zooxanthellaceae G.A.Klebs [76,77] and Blastodiniaceae Cavers [78] would have priority over Thoracosphaeraceae [79].

## 3. Brief Summary of Methods

The tree in Figure 1 is inferred from a “MAFFT” [80] generated nucleotide alignment (in total 2037 parsimony-informative positions). We defined the four regions of the ribosomal RNA (rRNA): SSU, ITS, LSU D1→D2, LSU D3→D10 and included all 199 Thoracosphaeraceae indicated by the bar on the right (plus 32 outgroup representatives), from which combinations of at least two loci were available. Additionally, we included sequences from *Paulsenella* Chatton that also show phylogenetic affinities to the Thoracosphaeraceae [59], but from which SSU data are only available. All outgroup taxa (other members of the Peridiniales, Amphidomataceae, Gymnodiniales) comprised the full sequence information. For the generation of new rRNA sequences from calcareous dinophyte strains out of our own culture collection (KF751921–KF751927), see the detailed descriptions in one of our previous studies [38]. Phylogenetic analyses were carried out using Maximum-Likelihood (ML) and Bayesian approaches, as described in detail previously [38]. The Bayesian analysis was performed using “MrBayes” v3.1.2 [81] under the GTR + Γ substitution model and the random-addition-sequence method with 10 replicates. We ran two independent analyses of four chains (one cold and three heated) with 15,000,000 cycles, sampled every 1000th cycle, with an appropriate burn-in (10%, after checking convergence). For the ML calculation, “RAxML” v7.2.6 [82] was applied by using the GTR + CAT substitution model to search for the best-scoring ML tree and a rapid bootstrap analysis of 1000 non-parametric replicates.
